# Spectral effects of light-emitting diodes on plant growth, visual color quality, and photosynthetic photon efficacy: White versus blue plus red radiation

**DOI:** 10.1371/journal.pone.0202386

**Published:** 2018-08-16

**Authors:** Yujin Park, Erik S. Runkle

**Affiliations:** Department of Horticulture, Michigan State University, East Lansing, Michigan, United States of America; National Research Council of Italy, ITALY

## Abstract

Arrays of blue (B, 400−500 nm) and red (R, 600−700 nm) light-emitting diodes (LEDs) used for plant growth applications make visual assessment of plants difficult compared to a broad (white, W) spectrum. Although W LEDs are sometimes used in horticultural lighting fixtures, little research has been published using them for sole-source lighting. We grew seedlings of begonia (*Begonia* ×*semperflorens*), geranium (*Pelargonium* ×*horturum*), petunia (*Petunia* ×*hybrida*), and snapdragon (*Antirrhinum majus*) at 20°C under six sole-source LED lighting treatments with a photosynthetic photon flux density (PPFD) of 160 μmol∙m^–2^∙s^–1^ using B (peak = 447 nm), green (G, peak = 531 nm), R (peak = 660 nm), and/or mint W (MW, peak = 558 nm) LEDs that emitted 15% B, 59% G, and 26% R plus 6 μmol∙m^−2^∙s^−1^ of far-red radiation. The lighting treatments (with percentage from each LED in subscript) were MW_100_, MW_75_R_25_, MW_45_R_55_, MW_25_R_75_, B_15_R_85_, and B_20_G_40_R_40_. At the transplant stage, total leaf area, and fresh and dry weight were similar among treatments in all species. Surprisingly, when petunia seedlings were grown longer (beyond the transplant stage) under sole-source lighting treatments, the primary stem elongated and had flower buds earlier under MW_100_ and MW_75_R_25_ compared to under B_15_R_85_. The color rendering index of MW_75_R_25_ and MW_45_R_55_ were 72, and 77, respectively, which was higher than those of other treatments, which were ≤64. While photosynthetic photon efficacy of B_15_R_85_ (2.25 μmol∙J^–1^) was higher than the W light treatments (1.51−2.13 μmol∙J^–1^), the dry weight gain per unit electric energy consumption (in g∙kWh^–1^) of B_15_R_85_ was similar to those of MW_25_R_75_, MW_45_R_55_, and MW_75_R_25_ in three species. We conclude that compared to B+R radiation, W radiation had generally similar effects on seedling growth at the same PPFD with similar electric energy consumption, and improved the visual color quality of sole-source lighting.

## Introduction

Light-emitting diodes (LEDs) are increasingly being used in the production of specialty crops (e.g., ornamental transplants and leafy greens) grown in controlled environments, including greenhouses and indoor vertical farms. When used indoors, sole-source lighting from LEDs enables one to tailor the radiation spectrum to elicit desirable plant growth attributes. Red (R, 600−700 nm) radiation is often considered the most efficient at driving photosynthesis based on the quantum yield from instantaneous leaf photosynthesis measurements [1]. Blue (B, 400−500 nm) radiation is added to R for normal photosynthetic functioning and to obtain desired phenotypes [2–4]. B and R LEDs also have the highest efficacy values in terms of photosynthetic photons emitted per watt of electricity [or photosynthetic photon efficacy (PPE) in μmol∙J^–1^] [5]. Thus, many commercial LED arrays developed for plant applications contain B and R LEDs. Diverse vegetable and floriculture crops have been grown successfully under B+R sole-source lighting, including lettuce (*Lactuca sativa*), tomato (*Solanum lycopersicum*), cucumber (*Cucumis sativus*), impatiens (*Impatiens walleriana*), salvia (*Salvia splendens*), petunia (*Petunia* × *hybrida*), vinca (*Catharanthus roseus*), geranium (*Pelargonium* × *hortorum*), and French marigold (*Tagetes patula*) [6–10].

One limitation of using B+R LEDs is that plants appear purplish to the human eye, causing difficulties in detecting nutritional deficiencies, disease symptoms, and physiological disorders. One possible solution is to add green (G, 500−600 nm) radiation to a B+R spectrum. Leaves absorb G radiation less effectively (by 16−23%) than B and R radiation [11], and two peaks of the relative quantum efficiency curve are in B and R radiation region [1]. Therefore, G radiation has been considered less efficient at driving photosynthesis than B or R radiation. However, the average relative quantum efficiency value for broadband G radiation is 0.87, which is slightly lower than that for R radiation (0.91) and higher than that for B radiation (0.73) [12]. In addition, while B and R radiation are strongly absorbed by chloroplasts in the upper part of the leaf, G radiation penetrates deeper into the leaf [13–15]. As the photosynthetic photon flux density (PPFD) increases, B and/or R photons saturate photosynthesis in the upper part of leaves, while penetrating G photons can be absorbed by chloroplasts in the lower part of leaves and thus, increase photosynthesis [14–15]. In general, at a constant PPFD, substituting B or R radiation with G radiation does not decrease plant growth. For example, shoot dry weight of impatiens, tomato, salvia, and petunia seedlings was similar under B+R (1:1) and under B+G (1:1) (at PPFD of 160 μmol∙m^–2^∙s^–1^) [16]. Similarly, in cherry tomato (*Lycopersicon esculentum*), plants grown under B+R (1:1) and B+R+G (3:3:1) had similar shoot dry weights (at PPFD of 320 μmol∙m^–2^∙s^–1^) [17]. In tomato, cucumber, pepper (*capsicum annum*), soybean (*Glycine max*), lettuce, and wheat (*Triticum aestivium*), substituting R radiation with G from 0% to 30% did not influence dry mass accumulation (at PPFD of 200 and 500 μmol∙m^–2^∙s^–1^) [18]. In addition, the substitution of 24% R radiation with G increased leaf area and dry weight (at PPFD of 150 μmol∙m^–2^∙s^–1^) [19]. Therefore, compared to B+R radiation, including G radiation in a sole-source lighting spectrum can have similar effects on plant growth while enabling people to more easily evaluate plant growth.

However, G LEDs are inefficient at converting electricity into photons (referred to as the “green gap”) and thus, adding G radiation from G LEDs is currently not practical. Another strategy is to use white (W) LEDs, alone or with R and/or B LEDs, to create a broad spectrum. W LEDs, which are created by adding phosphors to B LEDs to convert some of the B radiation to G and R, have a higher PPE value than G LEDs [6], and they emit a high portion of G radiation (e.g., 41 to 48%) [18]. Mixing these different narrow- and broad-band LEDs in various proportions enables control of the portions of B, G, and R radiation for both desirable human vision and plant growth responses.

The main considerations when determining the radiation spectrum of sole-source LED lighting have been its effects on plant growth attributes and its PPE. Meanwhile, a trade-off was made between the PPE of LEDs and its effects on human vision. Therefore, the challenge is to optimize the radiation spectrum for enhancing plant growth and PPE, while improving human vision performance. Here, we used mint white (MW) LEDs, which are rich in G radiation (59% of the PAR), and R LEDs to investigate the effects of different shades of W light on human vision, PPE, and plant growth and subsequent development of ornamental seedlings compared to a typical mixture of B+R radiation at the same PPFD. We postulated that compared to a typical B+R mixture, delivery of additional G radiation from MW LEDs would produce young plants with similar growth attributes while improving human vision, and with a minimal decrease in PPE. To evaluate the different colors of our lighting treatments and their effects on human vision, we quantified the correlated color temperature (CCT) and the color rendering index (CRI). We performed an experiment in highly controlled environments to evaluate plant growth attributes including fresh and dry weight, a variety of morphological traits, and subsequent flowering.

## Materials and methods

### Plant material

Begonia (*Begonia* × *semperflorens ‘*Olympia Red’), geranium (*Pelargonium* × *hortorum* ‘Deep Rose’), petunia (*Petunia* × *hybrida* ‘Wave Blue’), and snapdragon (*Antirrhinum majus* ‘Liberty Classic’) were selected for study because of their commercial significance and variations in shade tolerance and photoperiodic flowering response. Geranium, petunia, and snapdragon are shade-avoiding species while begonia is shade-tolerant. Geranium and begonia are day neutral; petunia and snapdragon are quantitative long-day plants. Seeds of each species were sown in 128-cell (2.7 × 2.7-cm; 12.0-mL volume) plug trays at a commercial greenhouse (C. Raker and Sons, Inc., Litchfield, MI). They were then transferred to research greenhouses at Michigan State University (East Lansing, MI) with a 16-h photoperiod at 20°C after the following number of days (rep. 1, 2): begonia (9, 17), geranium (9, 8), petunia (10, 9), and snapdragon (10, 9). The first true leaves emerged after the following number of days from seed sow (rep 1, 2): begonia (21, 19), geranium (9, 10), petunia (14, 14), and snapdragon (14, 14). Each plug tray was then cut into four sections (each with ≥30 seedlings), thinned to one plant per cell, and placed in each of six LED modules (semi-enclosed chambers) described by Wollaeger and Runkle [20].

### Radiation treatments and growth conditions

**Fig 1:**
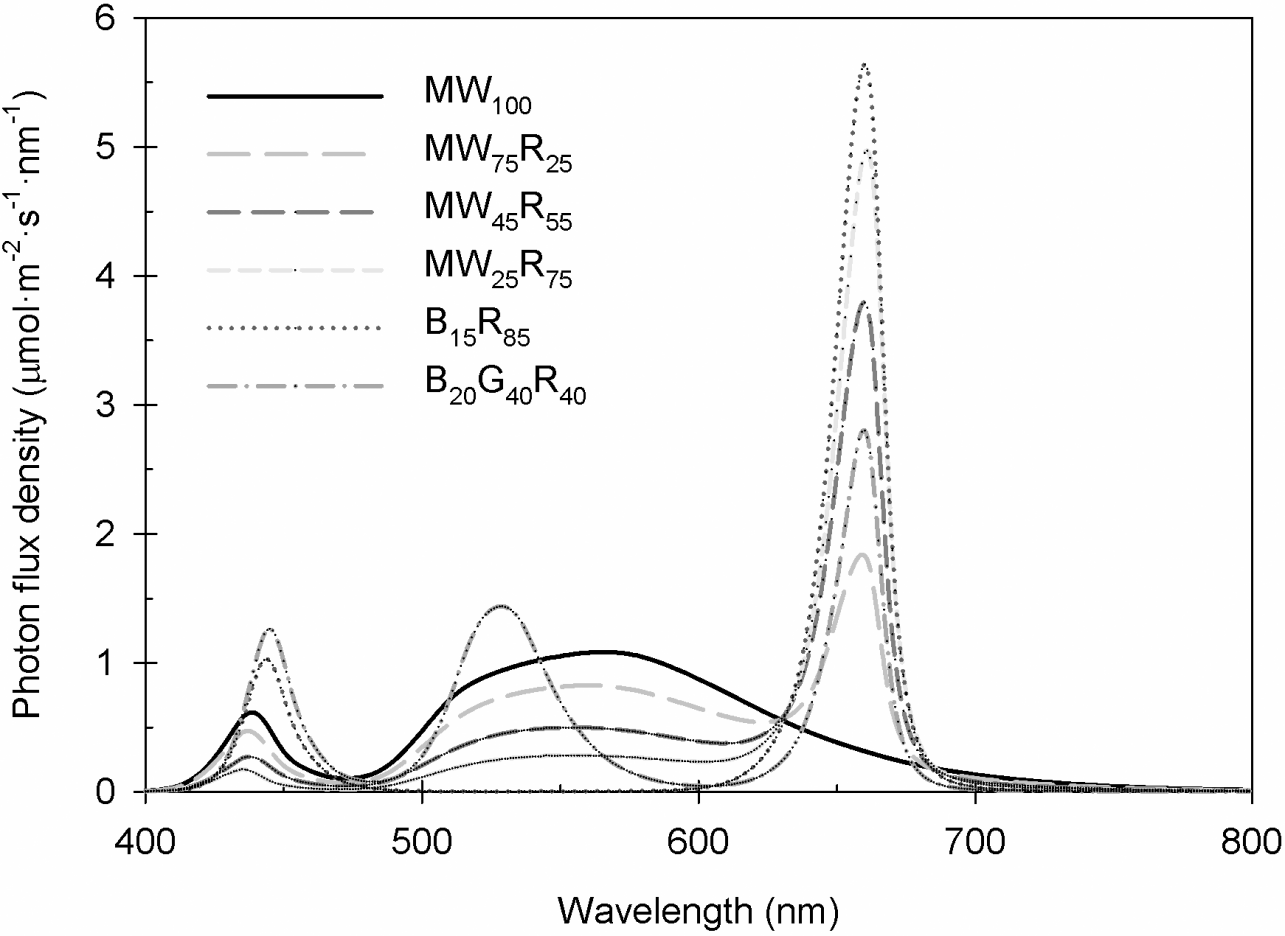
Spectral distribution of sole-source lighting treatments. Six sole-source lighting treatments were delivered from mint white (MW), red (R), blue (B), and green (G) light-emitting diodes (LEDs) at total photosynthetic photon flux density (PPFD) = 160 μmol∙m^−2^∙s^−1^. The subscript values after each LED type indicate the percentages of the total PPFD delivered from each LED type.

Six LED modules were located inside a refrigerated walk-in growth chamber at a constant temperature set point of 20°C. Each module was fitted with a new panel that contained 80 B (peak = 447 nm), G (peak = 531 nm), R (peak = 660 nm), and MW (peak = 558 nm) LEDs. The six treatments were designed to investigate the effects of MW with or without R and W radiation created with B, G, and R on plant growth compared to a typical B+R radiation spectrum (Fig 1). Each module delivered a PPFD of 160 μmol∙m^–2^∙s^–1^ that consisted of the following percentages: MW_100_ (100% PPFD from MW LEDs), MW_75_R_25_, MW_45_R_55_, MW_25_R_75_, B_20_G_40_R_40_, and B_15_R_85_. For each radiation treatment, the photon flux density of each LED type was adjusted manually by a dimmer switch on the driver boards based on an average of ten measurements on ten different predetermined horizontal positions inside each LED module at seedling-tray height using a spectroradiometer (PS-200; StellerNet, Inc., Tampa, FL). The photoperiod was 18 h [creating a daily light integral (DLI) of 10.4 mol∙m^–2^∙d^–1^] as controlled by a data logger (CR10; Campbell Scientific, Logan, UT). The plug trays were rotated daily inside each LED module to mitigate any positional effects. For each radiation treatment, the percentage of each waveband and R: far red (FR, 700−800 nm) was calculated using 100-nm wavebands; the phytochrome photostationary state (PSS) was estimated as described by Sager et al. [12]; and the yield photon flux density (YPFD), which is the product of photon flux density and relative quantum efficiency, was calculated based on McCree [1] and Sager et al. [12] (Table 1).

**Table 1:** Spectral characteristics of sole-source lighting treatments. Six sole-source lighting treatments were delivered from mint white (MW), red (R), blue (B), and green (G) light-emitting diodes (LEDs) at total photosynthetic photon flux density (PPFD) = 160 μmol∙m^−2^∙s^−1^. The subscript values after each LED type indicate the percentages of the total PPFD delivered from each LED type.

Radiation treatment	% B^a^	% G	% R	FR^b^	R:FR^c^	PSS^d^	YPFD^e^
MW_100_	15	59	26	6	8	0.84	141
MW_75_R_25_	11	45	44	4	17	0.87	142
MW_45_R_55_	7	27	66	3	35	0.88	145
MW_25_R_75_	4	16	80	2	57	0.88	146
B_15_R_85_	15	0	85	1	130	0.88	147
B_20_G_40_R_40_	20	40	40	1	108	0.87	134

^a^Percentage of B (400−500 nm), G (500−600 nm), and R (600−700 nm) radiation among total PPFD (400−700 nm).

^b^Photon flux integral of FR (700−800 nm) radiation in μmol∙m^−2^∙s^−1^.

^c^R:FR: Ratio of photon flux integral of R (600−700 nm) and FR (700−800 nm) radiation.

^d^PSS: phytochrome photostationary state following Sager et al. [12].

^e^YPFD: Yield photon flux density, which is the product of TPFD and relative quantum efficiency (in μmol∙m^−2^∙s^−1^) based on McCree [1] and Sager et al. [12].

**Fig 2:**
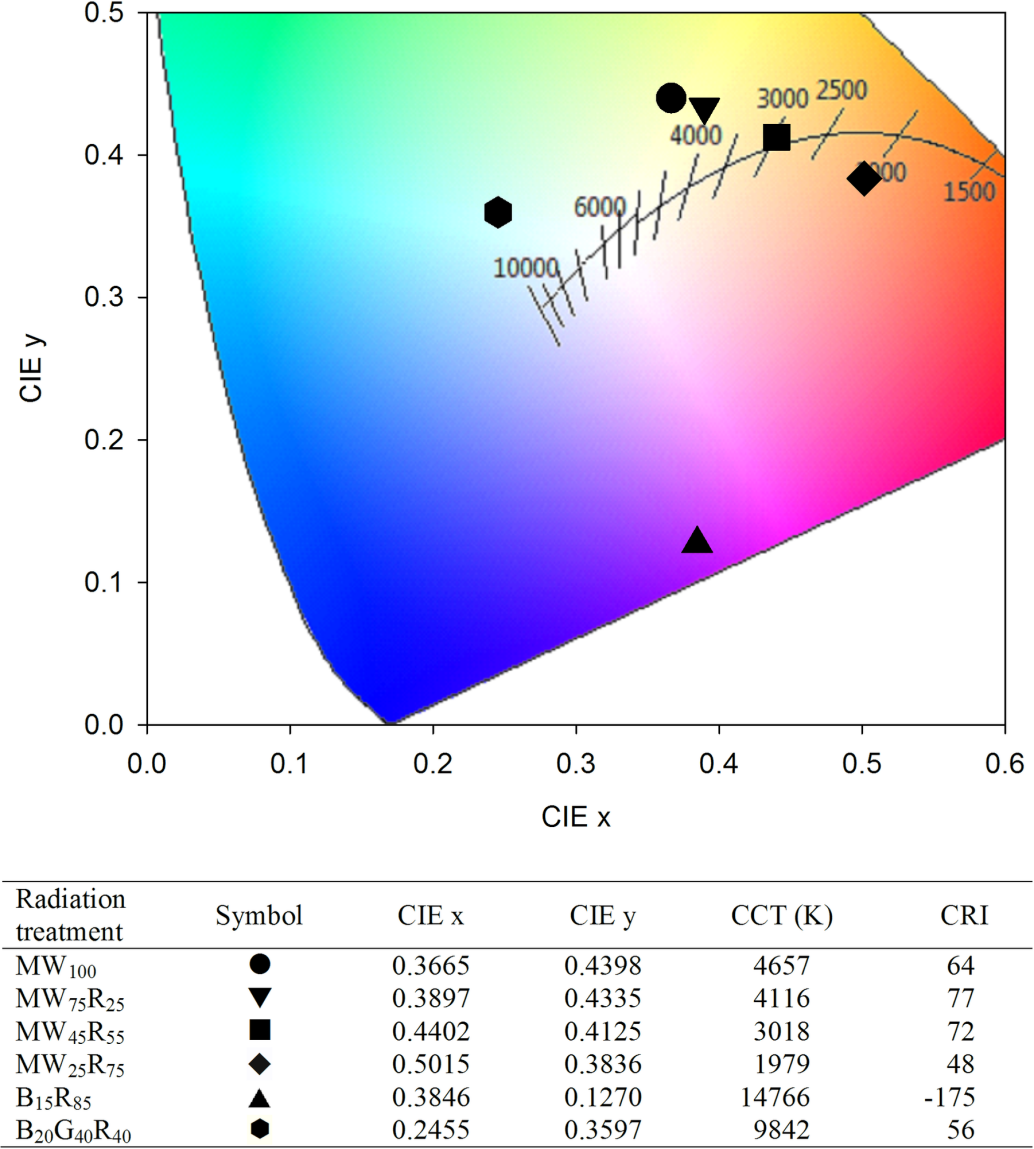
1931 CIE (x, y) chromaticity coordinates, correlated color temperature (CCT), and color rendering index (CRI) of sole-source lighting treatments. Six sole-source lighting treatments were delivered from mint white (MW), red (R), blue (B), and green (G) light-emitting diodes (LEDs) at a total photosynthetic photon flux density (PPFD) = 160 μmol∙m^−2^∙s^−1^. The subscript values after each LED type indicate the percentages of the total PPFD delivered from each LED type. CIE coordinates (x, y) for each sole-source lighting treatment (symbols) with the black-body curve (black solid line) and CCT values are presented.

The color of radiation treatments and their effects on vision for human eyes were characterized using the 1931 CIE (x, y) chromaticity coordinates and CCT, and for color performance with CRI. The 1931 CIE (x, y) chromaticity coordinates and CRI for each radiation treatment were determined by entering spectrum data into the ColorCalculator software (version 7.23; OSRAM Sylvania, Wilmington, NC, https://www.osram.us/cb/tools-and-resources/applications/led-colorcalculator/index.jsp) (Fig 2). The CCT for each radiation treatment was derived from the 1931 CIE (x, y) chromaticity coordinates using the following equation [21]: CCT (x, y) = −449n^3^ + 3525n^2^ − 6823.3n + 5520.33; where n = (x– 0.3320)/(y– 0.1858). The PPE of each LED type (at supply amperage of 450 mA) was 1.80, 0.54, 2.33, and 1.52 μmol∙J^–1^ for B, G, R, and MW LEDs (D. Hamby, OSRAM, personal communication in October, 2017), and the PPE of each radiation treatment was estimated by the product of the PPE of each LED type and the percentage of the total PPFD delivered by each LED type (Fig 2).

In each treatment, air and plant canopy temperature and radiation intensity were monitored and recorded as described by Park and Runkle [22]. Average air/canopy temperatures (°C) during the experimental periods were 21.1/21.0, 21.0/21.0, 21.1/21.2, 20.9/ 21.3, 20.5/20.7, and 21.0/21.3 for the MW_100_, MW_75_R_25_, MW_45_R_55_, MW_25_R_75_, B_15_R_85_, and B_20_G_40_R_40_ treatments, respectively. All temperatures had standard deviations (SD) ≤ ±1.0°C. Plants were irrigated as needed, every two or three days, through subsurface irrigation with deionized water supplemented with a water-soluble fertilizer providing (in mg∙L^-1^) 50 N, 19 P, 50 K, 23 Ca, 4 Mg, 1 Fe, 0.5 Mn, 0.5 Zn, 0.5 Cu, 0.3 B, and 0.1 Mo (MSU Plug Special; GreenCare Fertilizers, Inc., Kankakee, IL). The EC and pH of the nutrient solution was 0.43 mS∙cm^–1^ and 6.2, respectively.

### Data collection and analysis

The experiment was performed twice. In each replication, at the end of the seedling stage, ten random plants of each species in each treatment, usually excluding outer guard rows, were harvested the following number of days after seed sow (rep 1, 2): begonia (51, 52), geranium (33, 32), petunia (31, 34), and snapdragon (36, 37). The harvest times were determined when the seedlings were ready for transplanting (when the roots had grown sufficiently so that the seedlings could be easily pushed out of the trays with the entire root zone intact) and varied presumably because of differences in seed vigor.

The following data were collected on plants in each treatment: leaf (at node) number, total leaf area, stem length (from media surface to the apical meristem), and root and shoot fresh and dry weight. Petunia typically grows as a rosette and so its stem length was not measured at the transplant stage. A visible leaf that was ≥25% unfolded was counted in leaf number and included in leaf area. Total leaf area per plant was measured using a leaf-area meter (LI-3000; LI-COR, Lincoln, NE). Average individual leaf area was determined by dividing total leaf area by leaf number for each plant. For fresh and dry weight measurements, the shoot was excised at the medium surface, and the medium was carefully washed off to separate the roots. The shoots and roots were placed in separate envelopes and dried in an oven at ≥66°C for ≥5 d and weighed using an analytical balance (AB204-S; Mettler Toledo, Columbus, OH). Plant dry and fresh weight was determined by the sum of fresh and dry weight of shoot and root. Net assimilation was determined by dividing plant dry weight gain per unit leaf area for all species [18, 23].

For petunia, at the end of the seedling stage, 10 seedlings in each treatment were randomly selected and continuously grown under the LED modules until 59 d after seed sow for both replications. For both replications, date of the first visible bud was recorded for each plant and stem length was measured 53 d after seed sow.

Dry weight gain per unit electric energy consumption or dry weight efficacy (DWE) (in g∙kWh^–1^) for each radiation treatment and species in Table 2 was calculated using the following Eq (1):

$$DWE=\frac{DW\times N}{EEC}$$ (1)

where DW is dry weight (g) per plant for each radiation treatment and species; N is the total number of plants (128 seedlings) per LED module; and EEC is the electric energy consumption (kWh) per LED module (or each radiation treatment) for each species, which was estimated in the following Eq (2):

$$EEC=\frac{PP\times TH}{PPE}$$ (2)

where PP is the output of photosynthetic photons (μmol∙s^–1^) needed for the growing area in each LED module; TH is the total number of hours of sole-source lighting during the experiment, which was calculated by multiplying the photoperiod (18 h∙d^–1^) by the period of sole-source lighting treatments (34, 25, 24 and 19 d for begonia, geranium, snapdragon, and petunia, respectively); PPE is photosynthetic photon efficacy (μmol∙J^–1^ or μmol∙s^–1^∙W^–1^), which was calculated for each radiation treatment (Table 2) using the following the Eq (3);

$$PP=\frac{PPFD\times A}{PUE}$$ (3)

where PPFD is the PPFD for the sole-source lighting treatments (160 μmol∙m^–2^∙s^–1^); A is the growing area (or the bottom surface area) of each LED module (0.8 m × 0.27 m = 0.216 m^2^); and PUE is the photon use efficiency of the LED module, which indicates the proportion of the photon flux received by the growing area (or the bottom surface) compared to that emitted by the LED fixtures. PUE depends on the properties of the LED fixture, such as beam distribution, reflector design, and geometries [24]. In this study, considering the highly reflective walls of each LED module, the PUE was estimated as 0.9.

**Table 2:** Photosynthetic photon efficacy (PPE) and dry weight gain per unit electric energy consumption (dry weight efficacy) for four species. Begonia ‘Olympia Red’, geranium ‘Pinto Premium Deep Rose’, snapdragon ‘Liberty Classic Yellow’, and petunia ‘Wave Blue’ seedlings were grown for 34 d, 25 d, 24 d and 19 d, respectively, under six sole-source lighting treatments delivered from mint white (MW), red (R), blue (B), and green (G) light-emitting diodes at a total photosynthetic photon flux density (PPFD) = 160 μmol∙m^−2^∙s^−1^. The values after each LED type represent their percentages of the total PPFD.

Radiation treatment	PPE (μmol∙J^–1^)	Dry weight efficacy (g∙kWh^–1^)			
		Begonia	Geranium	Snapdragon	Petunia
MW_100_	1.52	0.78 b^a^	2.18 cd	1.37	1.00 cd
MW_75_R_25_	1.72	0.92 ab	2.44 bcd	1.53	1.12 bcd
MW_45_R_55_	1.88	1.17 a	2.70 bc	1.58	1.30 abc
MW_25_R_75_	2.13	1.11 a	3.52 a	1.78	1.50 a
B_15_R_85_	2.25	1.14 a	3.02 ab	1.76	1.36 ab
B_20_G_40_R_40_	1.51	0.79 b	1.88 d	1.32	0.91 d
Significance		**^b^	**	NS	**

^a^Means with different letters are significantly different by Tukey’s honestly significant difference test (*P* < 0.05) and lack of mean separation indicates nonsignificance.

^b^NS or ** Nonsignificant or significant at *P* < 0.01, respectively.

The experiment used a randomized complete block design with two blocks and ten subsamples per block. Each replication was regarded as a block. Each LED module was regarded as the experimental unit for the radiation treatment. Within each LED module, ten individual seedlings per species were the sub-samples or observational units. Data were analyzed with SAS (version 9.4; SAS Institute, Cary, NC) using the PROC MIXED procedure [with a fixed factor for radiation treatments, a random factor of blocks (or replications), and a random factor for interaction between blocks and radiation treatments] that provided pairwise comparisons between treatments using Tukey’s honestly significant test at *P* ≤ 0.05. Regression analysis was performed with the PROC REG procedure to investigate the correlation between net assimilation and the estimated YPFD of the sole-source lighting treatments. In regression analysis, the mean for each replication was treated as a single data point and included 12 data points for the six radiation treatment effects (2 replications× 6 treatments).

## Results and discussion

### Visual and color properties

CRI evaluates the accuracy of light sources to render human color perception of objects compared to a reference light source (including black body radiation for light sources having CCT <5000 K, or natural daylight for those having CCT ≥5000 K) [25–26]. The maximum value of CRI is 100, and a light source with a CRI ≥80 is typically considered good at rendering the color of objects and elicits a comfortable human visual perception [27]. For example, typical CRI values of common light sources include 100 for incandescent lamps, 89 for fluorescent lamps, and 24 for high-pressure sodium lamps [28]. In this study, the CRI value of B_15_R_85_ was negative, while MW_100_ had a CRI value of 64 and substituting MW with R LEDs by 25% or 55% increased the CRI to 77 or 72, respectively (Fig 2). However, when 75% of MW was substituted with R, the CRI value decreased to 47, which was lower than B_20_G_40_R_40_ (CRI = 56). The CRI values for all of our W light treatments were lower than the recommended CRI value ≥80, but were much higher than a typical B+R spectrum used in horticulture.

The color appearance of light emitted by a light source can be described with CCT [29]. CCT is the absolute temperature of a blackbody radiator, expressed in degrees Kelvin (K), whose chromaticity is closest to that of the light source [28, 30]. Based on the CCT value, W light can be categorized as warm W (2500−3500 K), neutral W (3500−4500 K), and cool W (4500−5500 K) [26]. The MW LEDs used in this study had a CCT of 4657 K and thus can be described as cool W. As MW was increasingly substituted with R, the CCT decreased to 1979 K (Fig 2). Thus, the W light created by MW_75_R_25_ (4116 K) and MW_45_R_55_ (3018 K) can be categorized into neutral W and warm W, respectively. In addition, the CCT of MW_45_R_55_ was within the recommended CCT ranges (2700−4000K) for a natural color perception [26]. The CCT values for B_15_R_85_ (14766 K) and B_20_G_40_R_40_ (9842 K) were outside of the range of W light.

### Plant growth and development

**Fig 3:**
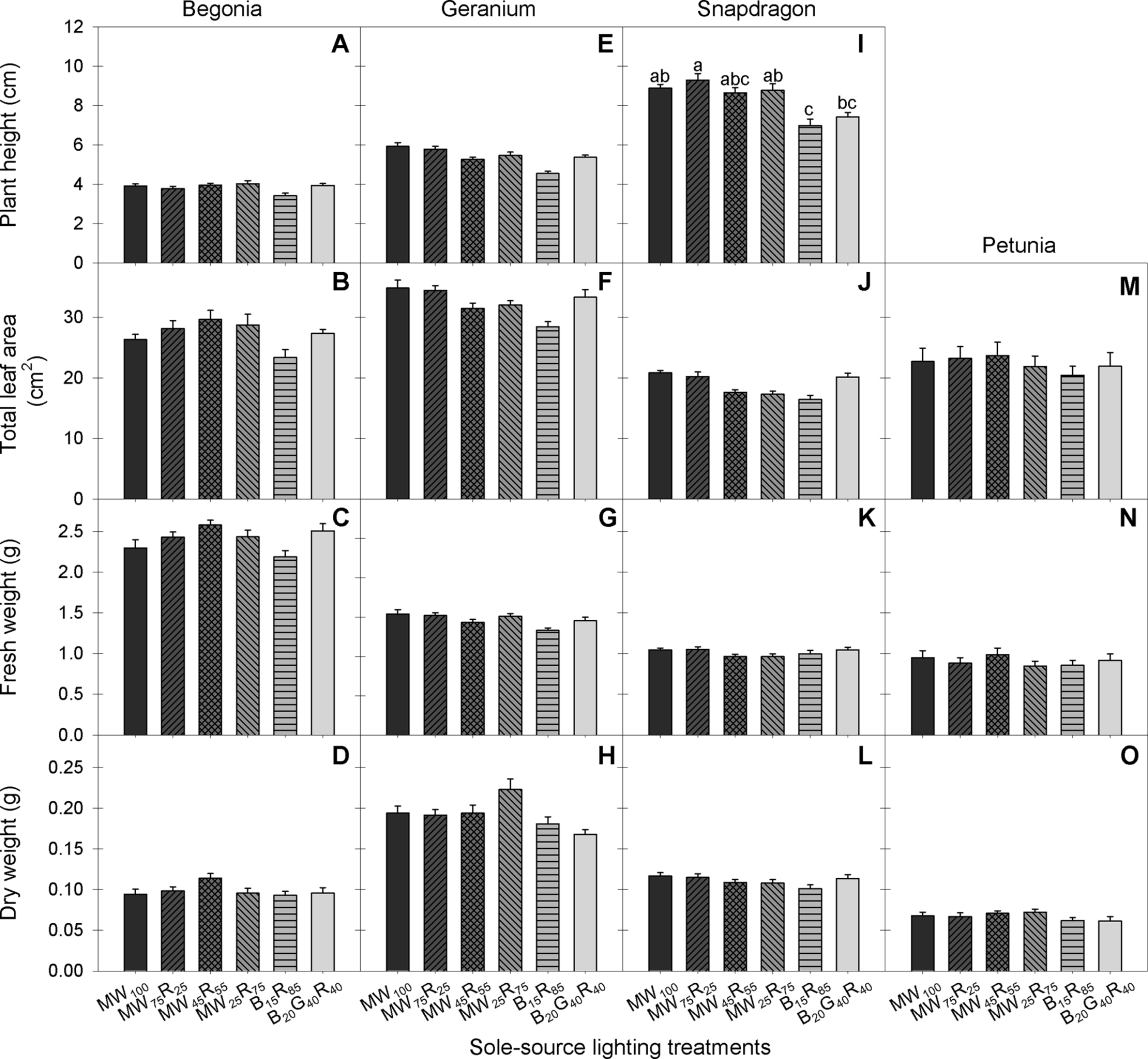
Plant height, total leaf area, fresh and dry weight for four species at the transplant stage (A-O). Begonia ‘Olympia Red’, geranium ‘Pinto Premium Deep Rose’, snapdragon ‘Liberty Classic Yellow’, and petunia ‘Wave Blue’ seedlings were grown for 34 d, 25 d, 24 d and 19 d, respectively, under six sole-source lighting treatments delivered from mint white (MW), red (R), blue (B), and green (G) light-emitting diodes at a total photosynthetic photon flux density (PPFD) = 160 μmol∙m^−2^∙s^−1^. The values after each LED type represent their percentages of the total PPFD. Means with different letters are significantly different by Tukey’s honestly significant difference test (*P* < 0.05) and lack of mean separation indicates nonsignificance. Error bars indicate standard errors of 20 observational units [two replications with 10 subsamples (plants) per replication per species].

**Fig 4:**
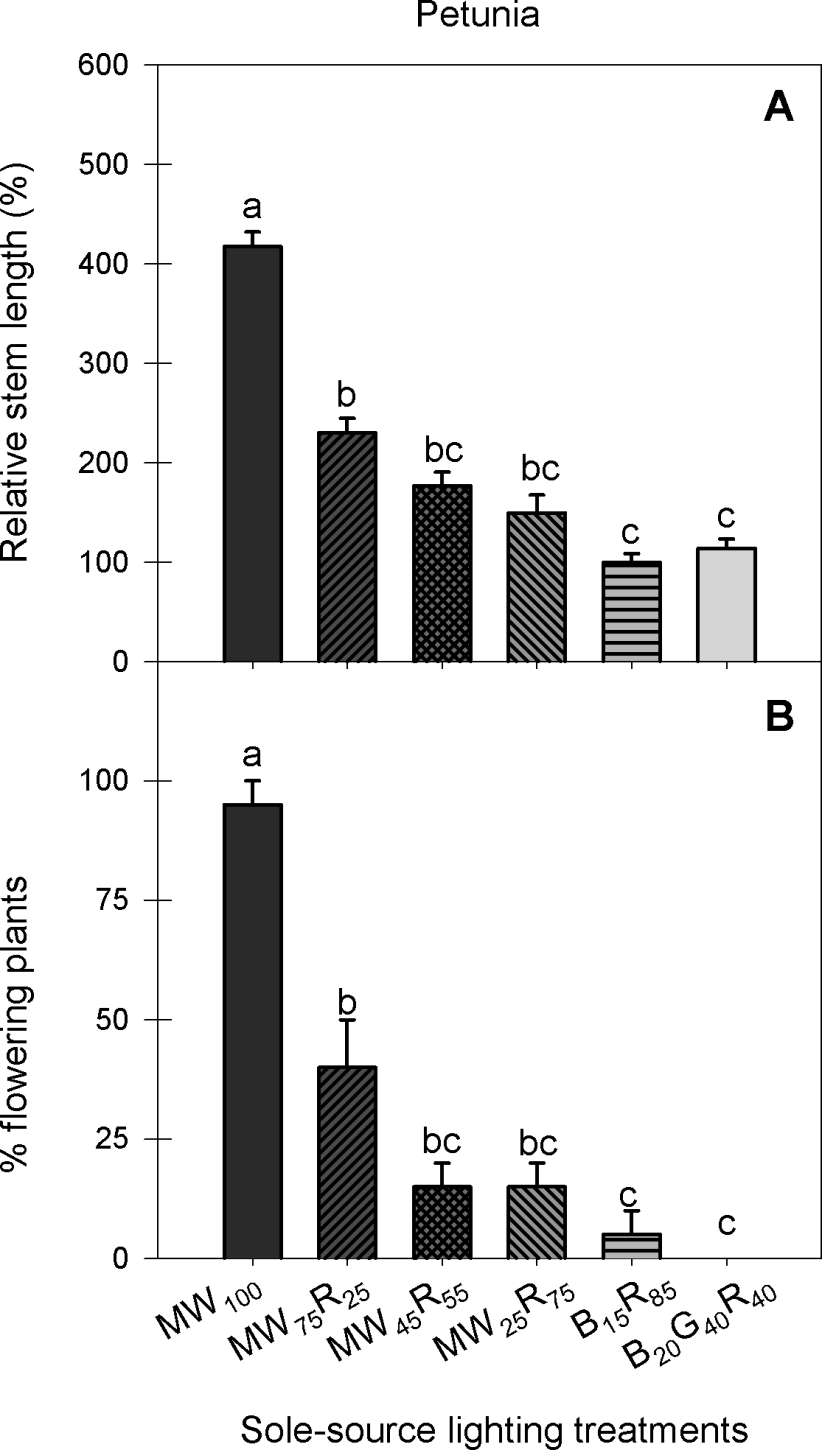
Stem length and percentage of flowering plants for petunia after the transplant stage. Stem length (**A**) and percentage of flowering plants (**B**) of petunia ‘Wave Blue’ grown for 39 d and 45 d, respectively, under six sole-source lighting treatments delivered from mint white (MW), red (R), blue (B), and green (G) light-emitting diodes at a total photosynthetic photon flux density (PPFD) = 160 μmol∙m^−2^∙s^−1^. The values after each LED type represent their percentages of the total PPFD. Stem length is presented relative to that of the plants grown under the B_15_R_85_ treatment. Percentage of flowering plants was calculated by dividing the number of plants that had visible buds by that of the total number of plants for each radiation treatment. Means with different letters are significantly different by Tukey’s honestly significant difference test (*P* < 0.05). Error bars for relative stem length and percentage of flowering plants indicate standard errors of 20 observational units [two replications with 10 subsamples (plants) per replication and species (n = 20)] and two replications (n = 2), respectively.

In general, seedling growth characteristics, including plant height, total leaf area, and fresh and dry weight of all species tested in this study were similar among the different shades of W radiation and B+R radiation at the same PPFD, except for seedling height in snapdragon (Fig 3). In snapdragon, seedlings grown under MW_100_, MW_75_R_25_ and MW_25_R_75_ were 26−33% taller than those grown under B_15_R_85_. Similarly, when petunia seedlings were grown longer (beyond the transplant stage) under the sole-source lighting treatments, the primary stem elongated and had flower buds earlier under MW_100_ and MW_75_R_25_ compared to under B_15_R_85_ (Fig 4). The differences in radiation spectrum between MW_100_ and MW_75_R_25_ and B_15_R_85_ treatments include a higher portion of G radiation and a lower R:FR (Table 1), both of which can affect stem elongation and flowering time.

Plants absorb most PAR (400−700 nm), especially B and R radiation, but they transmit and reflect most FR radiation. Therefore, under a plant canopy, the R:FR is decreased. Plants detect a reduction in the R:FR through phytochrome photoreceptors, which trigger shade-avoidance responses including promotion of extension growth [31–32]. For example, in the shade-avoiding geranium, petunia, and snapdragon, stem length increased linearly as the R:FR decreased from 1:0 to 1:1 [22]. The R:FR is also involved in regulating photoperiodic flowering, and an intermediate R:FR of photoperiodic lighting during the finishing stage and sole-source lighting during the seedling stage accelerated flowering in some long-day plants [22, 33–34]. In this study, MW_100_ and MW_75_R_25_ emitted a small amount of FR radiation (4−6 μmol∙m^–2^∙s^–1^) and thus had a lower R:FR (8:1−17:1) value than other treatments (Table 1). Thus, the stem elongation and flowering promotion under MW_100_ and MW_75_R_25_ could be influenced by a lower R:FR.

In addition, a few studies showed that G radiation, similar to FR radiation, induces shade-avoidance responses, including elongated hypocotyls and petioles [35–37]. Genetic studies have demonstrated that G radiation reverses B radiation-mediated inhibition of hypocotyl and stem elongation by inactivating cryptochromes [38–40]. In addition, while the effects on G radiation on flowering are still less clear, the heading of wheat was promoted as the portion of G radiation in the radiation spectrum increased [41]. In this study, the percentage of G radiation (in PAR) of MW_100_ and MW_75_R_25_ was 59% and 45%, respectively while B_15_R_85_ had 0% of G radiation (Table 1). Thus, the stem elongation and flowering promotion under MW_100_ and MW_75_R_25_ could be at least partly attributed an increasing percentage of G radiation in the MW_100_ and MW_75_R_25_ treatments. Because of the possible interactions of G radiation, the R:FR ratio, and other differences in the spectrum, we cannot attribute the stem elongation and flowering promotion responses under the MW_100_ and MW_75_R_25_ treatments to specific spectral components.

In plant growth analysis, total leaf area and net assimilation determines plant dry weight gain [18, 23, 42]. Light quality can influence plant dry mass accumulation by altering leaf expansion and by affecting photosynthesis associated with the wavelength dependence of the quantum yield [5, 43]. YPFD has been used to quantify the effects of light quality on photosynthesis [12, 44]. In this study, while MW+R treatments promoted stem elongation in snapdragon and petunia and flowering in petunia compared to B_15_R_85_, spectral differences of lighting treatments had negligible effects on total leaf area in all species (Fig 3). In addition, because G radiation has a lower average relative quantum efficiency value (0.85) than R radiation (0.91), the calculated YPFD was 1−4% lower in the MW+R treatments and 9% lower in B_20_G_40_R_40_ treatment than B_15_R_85_ (Table 1). However, whole-plant net assimilation did not correlate well with the calculated YPFD of sole-source lighting treatments and the marginally lower YPFD of the MW+R and B_20_G_40_R_40_ treatments had little to no effect on whole-plant net assimilation in any species (data not shown). With few significant spectral effects of lighting treatments on leaf expansion and net assimilation, plant dry weight was similar among radiation treatments in all species (Fig 3).

The relative quantum efficiency curve represents the direct effects of the radiation spectrum on instantaneous photosynthesis per unit of leaf area [1]. However, when plants are grown under a particular radiation spectrum over time, they can acclimate by altering the relative size of the photosystems, chlorophyll composition, and chlorophyll content [43, 45–46]. Photosynthetic acclimation to the radiation spectrum optimizes electron transport and improves photosynthetic efficiency [47]. Therefore, photosynthetic acclimation at least partially diminishes the direct effects of YPFD on instantaneous photosynthesis, resulting in no clear correlation between YPFD of each radiation spectrum and whole plant net assimilation. This is supported by several long-term studies in which net assimilation was not correlated with the estimated YPFD [48–49]. Together, these results indicate that YPFD is not necessarily a predictive indicator of long-term effects of the radiation spectrum on photosynthesis and plant growth.

### Photosynthetic photon efficacy (PPE)

PPE, which describes the PAR photon output per electric energy input (in μmol∙J^–1^), is considered as the appropriate metric for electrical efficiency of light sources for plant growth [6, 29, 50]. In this study, B_15_R_85_ had the highest PPE value of 2.25 μmol∙J^–1^ (Table 2). The PPE of MW_100_ was 1.52 μmol∙J^–1^, which was 33% lower than that of B_15_R_85_, but as more MW was substituted with R, the PPE increased to 2.13 μmol∙J^–1^. B_20_G_40_R_40_ had a comparable PPE (1.51 μmol∙J^–1^) as the MW_100_. We also calculated dry weight gain per unit electric energy consumption, or DWE (in g∙kWh^–1^). While B_15_R_85_ had a higher PPE (in μmol∙J^–1^) than the W light treatments, the DWE of B_15_R_85_ was similar with those of MW_25_R_75_, MW_45_R_55_, and MW_75_R_25_ in begonia, petunia, and geranium (Table 2). Only DWE for MW_100_ and B_20_G_40_R_40_ was 26−38% lower than that of B_15_R_85_ in those three species. In snapdragon, DWE was similar among light treatments.

Here, we evaluated different shades of W and B+R radiation in terms of their effects on plant growth, electrical efficiency, and visual and color qualities. Seedling growth was similar under B+R and W radiation treatments at the same PPFD. In addition, W radiation created by mixing MW and R LEDs produced plant dry mass as efficiently as a typical B+R mixture. Using W radiation generally increased the visual quality (or CRI value), and particularly, some mixtures of MW and R LEDs showed higher visual quality and optimum color quality for W light. These results suggest that W radiation can be used for sole-source lighting to produce young plants with similar growth attributes and electric energy consumption while improving human vision.

## Abbreviations

B: blue radiation
CCT: correlated color temperature
CRI: color rendering index
DLI: daily light integral
DW: dry weight
DWE: dry weight efficacy
FR: far-red radiation
G: green
LEDs: light-emitting diodes
MW: mint white
PAR: photosynthetically active radiation
PPE: photosynthetic photon efficacy
PPFD: photosynthetic photon flux density
PSS: phytochrome photostationary state
R: red
W: white
YPFD: yield photon flux density
